# Inhibition of herpes simplex virus 1 gene expression and replication by RNase P-associated external guide sequences

**DOI:** 10.1038/srep27068

**Published:** 2016-06-09

**Authors:** Jin Liu, Luyao Shao, Phong Trang, Zhu Yang, Michael Reeves, Xu Sun, Gia-Phong Vu, Yu Wang, Hongjian Li, Congyi Zheng, Sangwei Lu, Fenyong Liu

**Affiliations:** 1 State Key Laboratory of Virology, College of Life Sciences, Wuhan University, Wuhan, Hubei 430072, China; 2 School of Public Health, University of California, Berkeley, CA 94720, USA; 3 Taizhou Institute of Virology, Taizhou, Jiangsu 225300, China; 4 Jiangsu Affynigen Biotechnologies, Inc., Taizhou, Jiangsu 225300, China; 5 College of Life Sciences, Jinan University, Guangzhou, Guangdong 510632, China

## Abstract

An external guide sequence (EGS) is a RNA sequence which can interact with a target mRNA to form a tertiary structure like a pre-tRNA and recruit intracellular ribonuclease P (RNase P), a tRNA processing enzyme, to degrade target mRNA. Previously, an *in vitro* selection procedure has been used by us to engineer new EGSs that are more robust in inducing human RNase P to cleave their targeted mRNAs. In this study, we constructed EGSs from a variant to target the mRNA encoding herpes simplex virus 1 (HSV-1) major transcription regulator ICP4, which is essential for the expression of viral early and late genes and viral growth. The EGS variant induced human RNase P cleavage of ICP4 mRNA sequence 60 times better than the EGS generated from a natural pre-tRNA. A decrease of about 97% and 75% in the level of ICP4 gene expression and an inhibition of about 7,000- and 500-fold in viral growth were observed in HSV infected cells expressing the variant and the pre-tRNA-derived EGS, respectively. This study shows that engineered EGSs can inhibit HSV-1 gene expression and viral growth. Furthermore, these results demonstrate the potential for engineered EGS RNAs to be developed and used as anti-HSV therapeutics.

Herpes simplex virus 1 (HSV-1), a member of the human herpesvirus family, is one of the leading causes of viral infections in humans and reactivation of the virus from latency can cause herpes simplex encephalitis and corneal blindness in immunocompromised individuals^1^. It is necessary to develop new antiviral compounds and novel approaches because of the emerging drug-resistant strains of HSV-1. Nucleic acid based molecules represent promising therapeutic strategies for targeting of specific mRNA sequences^2^. Ribonuclease P (RNase P) is a ribonucleoprotein complex which facilitates the maturation of tRNA by catalyzing a hydrolysis reaction to remove the leader sequence of precursor tRNA (pre-tRNA) (Fig. 1A)^2^,^3^,^4^. It has been shown by previous studies that RNase P recognizes pre-tRNAs with their tertiary molecular structures, and can cleave a custom-designed substrate which resembles a pre-tRNA in tertiary structure^5^. In seminar studies by Altman and colleagues, targeted mRNAs were able to be cleaved by recruited RNase P when bound to custom-designed EGSs to form a hybrid resembling a pre-tRNA molecule (Fig. 1B)^6^,^7^. EGSs which were derived from natural pre-tRNA sequences can be used to inhibit gene expression in both bacterial and mammalian cells^7^,^8^,^9^,^10^. Furthermore, EGSs were shown to be effective in inhibiting gene expression and replication of several human viruses including HIV, human cytomegalovirus (HCMV), Kaposi sarcoma-associated herpesvirus (KSHV), and hepatitis B virus (HBV) in human cultured cells^11^,^12^,^13^,^14^.

The technology of EGS-induced mRNA cleavage by RNase P is distinguished by its simple mechanism from other nucleic acid approaches for mRNA silencing such as RNAi, which involves various cellular factors. Furthermore, the RNase P-mediated cleavage does not generate nonspecific “irrelevant cleavage” which happens in RNase H-mediated cleavage induced by conventional antisense phosphothioate molecules^3^,^15^. Therefore, EGSs have the potential to be an effective therapeutic approach for human diseases such as HSV-1 infection^16^,^17^,^18^.

Increasing both the *in vitro* and *in vivo* activity of EGSs can contribute to the efficiency of the reaction of EGS-induced cleavage of target RNA by RNase P. In a previous study, novel EGS variants which were more efficient in recruiting RNase P in the cleavage of HSV-1 thymidine kinase (TK) mRNA were screened out with an *in vitro* selection procedure^19^. As of now, little is known about the mechanistic changes in these EGS RNA variants that improve their activity in inducing the cleavage of a target mRNA by RNase P and whether they are also effective in targeting other HSV mRNAs to influence the expression of viral genes and further affect viral growth.

In this study, several EGSs were constructed to target the mRNA encoding HSV-1 ICP4 protein. ICP4, a viral immediate early (IE) protein functioning as a viral major transcription activator, is essential for the expression of viral β (early) and γ (late) genes and viral replication^1^. We determined the activity of the constructed EGSs in guiding RNase P to cleave the target HSV-1 ICP4 mRNA sequence and their efficacy in inhibiting HSV-1 gene expression and viral replication in HSV infected cells. C468-A, an EGS variant, showed 60 times more efficient in guiding RNase P to cleave ICP4 mRNA sequence than SER-A derived from a natural pre-tRNA sequence. C468-A is more effective than SER-A in the inhibition of the mRNA and protein expression of ICP4 when expressed in HSV infected cells. Cells that expressed C468-A showed a decrease of about 97% in ICP4 gene expression and an inhibition of about 7,000 times in HSV-1 growth. In comparison, cells expressing no EGS or a control non-functional EGS only showed less than 10% reduction in the expression levels of viral genes and no significant effect on viral growth. Our results demonstrate that EGSs are effective in inhibiting HSV-1 growth in cultured cells.

## Results

### EGS-mediated cleavage of ICP4 mRNA sequence by RNase P *in vitro*

We determined the accessible regions of ICP4 mRNA in HSV-1 infected cells with a dimethyl sulphate (DMS)-based mapping approach^19^,^20^,^21^. HSV-1 infected HeLa cells were incubated in media containing DMS. DMS can modify the nucleotides of the accessible mRNA regions after entering the cells. Total mRNA from these cells was isolated and primer extension assays with reverse transcriptase were used to determine the regions modified by DMS, which were also the accessible regions in the ICP4 mRNA sequence. A position, 66 nts downstream from the start codon was chosen as the cleavage site for the EGSs since it was among the regions where the ICP4 mRNA was most modified by DMS.

**Figure 1:**
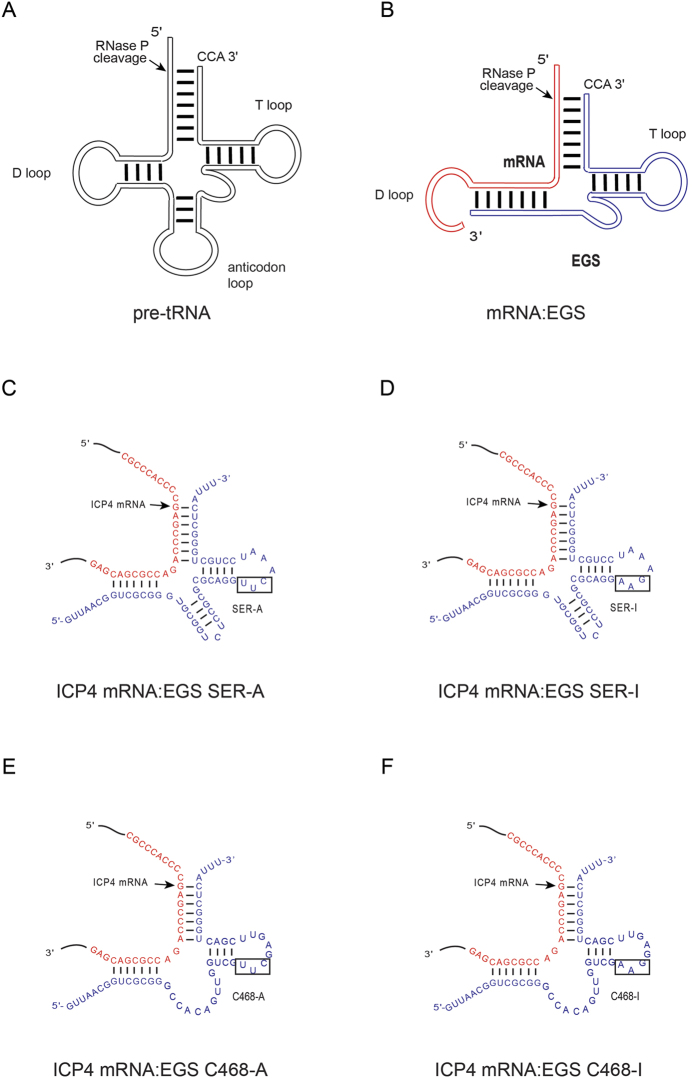
Schematic presentation of RNase P substrate/synthesized EGS complex. (**A**) A pre-tRNA structure. (**B**) A hybridized complex of a target RNA and an EGS resembling the T-stem and loop, and variable region of a pre-tRNA structure. (**C–F**) Hybridized complexes of HSV-1 ICP4 mRNA and EGS SER-A, SER-I, C468-A, and C468-I, respectively. The sequences of SER-A and SER-I were derived from natural pre-tRNA^SER^, while those of C468-A and C468-I were from EGS variant C468. The targeting sequences of ICP4 mRNA are shown in red and the EGS sequences are shown in blue, respectively. The site of cleavage by RNase P is marked with an arrowhead. The three mutated positions to inactivate EGS activity are marked in black box.

Previously, we carried out an *in vitro* selection procedure to isolate EGS variants which were more active in guiding RNase P to cleave HSV-1 TK mRNA than the EGSs derived from a natural pre-tRNA^19^. The original goal of the research was to construct effective gene-targeting EGSs and find out how EGSs guide RNase P to cleave target RNA by studying those variants. However, how these screened-out EGSs gain enhanced activity in guiding RNase P to cleave target mRNA sequences is still unknown. Among those most active EGSs in guiding RNase P to cleave target mRNA sequences, C468 efficiently recruited RNase P to cleave both HSV-1 TK mRNA and ICP4 mRNA (see below, Table 1). EGS C468-A was designed by attaching the T loop and variable domain of C468 to parts of complementary sequence of the ICP4 mRNA (Fig. 1E). Another EGS, SER-A, which was derived from the natural pre-tRNA^SER^ sequence, was constructed in the same way as C468-A (Fig. 1C).

**Figure 2:**
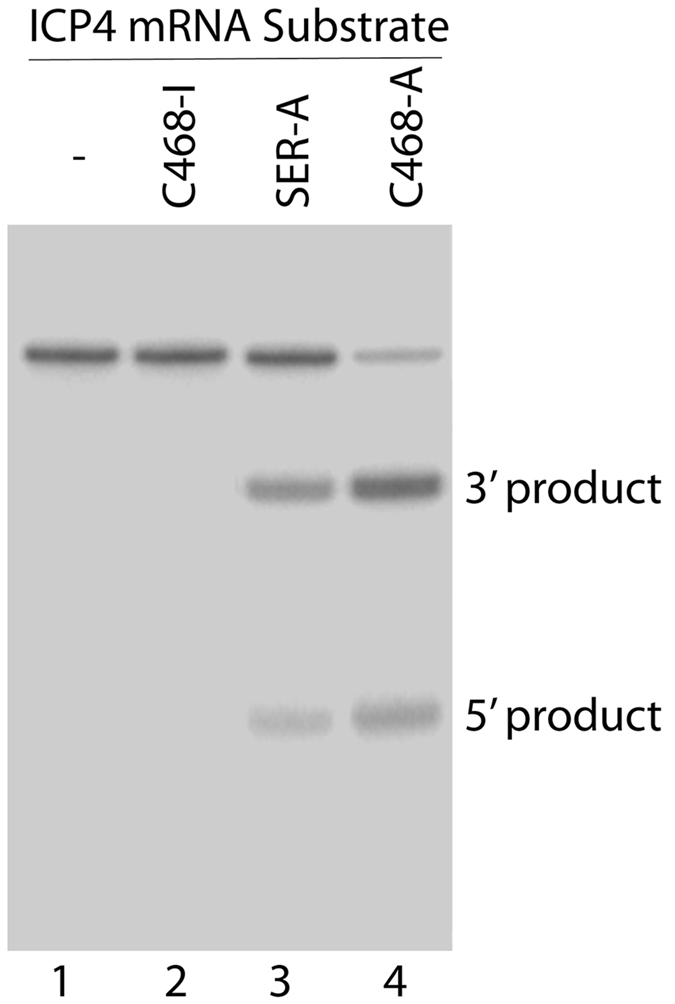
*In vitro* cleavage of an ICP4 mRNA substrate by human RNase P. The reactions were carried out in the absence of an EGS (-, lane 1) and presence of different EGSs (lanes 2–4).

We measured the RNase P mediated cleavage rate of substrate icp38 containing 38 nucleotides of ICP4 mRNA, which represents the target sequence, in the presence of C468-A or SER-A. Efficient cleavage of icp38 by human RNase P was observed in the presence of active EGS SER-A and C468-A (Fig. 2, lanes 3–4). Applying kinetic analyses, we obtained the cleavage efficiency values [V_max(apparent)_/K_m(apparent)_] for the cleavage reactions. C468-A was 60 times more efficient in guiding human RNAse P to cleave icp38 than SER-A (Table 1). It is possible that the variable loop of C468-A contributes to the stability of the mRNA-EGS complex and thus improves RNase P cleavage rate. In that case, the binding affinity of C468-A to the target ICP4 mRNA sequence should be better than that of SER-A. Using gel-shift assays, the binding affinities of C468-A and SER-A to icp38 RNA sequence were measured and represented by the dissociation constant (K_d_). Despite that C468-A and SER-A share the same complementary sequence of the ICP4 mRNA (Fig. 1C,E), C468-A showed about 80 times higher binding affinity to icp38 than SER-A (Table 1). These results imply that the enhanced binding affinity of C468 A to icp38 may be due to newly introduced tertiary interactions.

C468-I and SER-I, derived from C468-A and SER-A respectively, were designed and used as negative controls. These two EGSs contain the same mutations (from 5′-UUC-3′ to AAG) in their T- loop compared with C468-A or SER-A (Fig. 1D,F). The mutated nucleotides are highly conserved and changes at this site inactivate EGS activity^3^. The two control EGSs showed targeting activities at least 2 × 10^3^-fold slower than C468-A (Table 1; Fig. 2, lane 2). C468-I and SER-I contained the identical complementary sequence to ICP4 mRNA sequence with C468-A or SER-A (Fig. 1C–F), and also showed similar binding affinities to icp38 with C468-A and SER-A, respectively (Table 1). Thus, C468-I and SER-I, which share the similar structures with C468-A and SER-A respectively but with inactivated EGS activity, can serve as controls to exclude antisense or other unspecific effect of EGSs.

### The expression of EGSs in human cells

**Figure 3:**
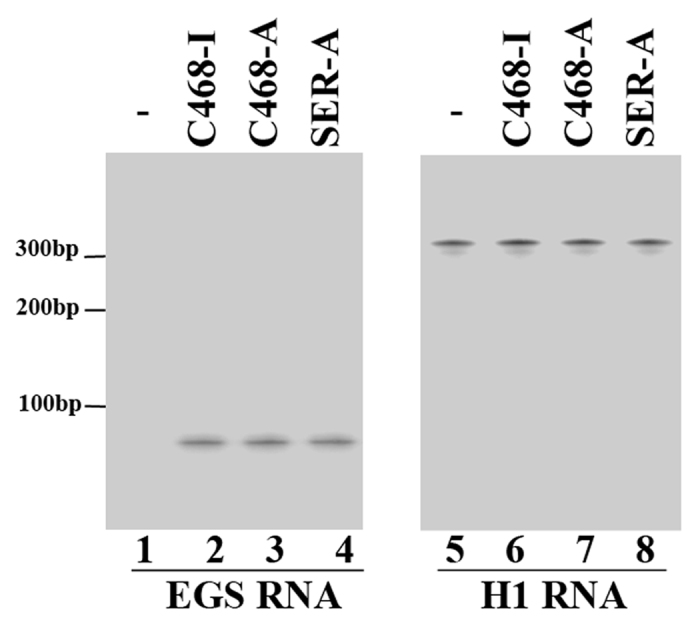
EGS RNA expression with Northern blot analysis in HeLa cells. RNA samples (25 μg) were isolated from parental HeLa cells (-, lanes 1 and 5) and cells expressing EGS C468-I (lanes 2 and 6), C468-A (lanes 3 and 7), and SER-A (lanes 4 and 8). Human H1 RNA (lanes 5–8) was used as the loading control.

To construct HeLa and human primary oral keratinocyte (HOK) cell lines expressing EGSs, DNA sequences encoding SER-I, SER-A, C468-I, and C468-A were cloned into retroviral vector LXSN under the control of the small nuclear U6 RNA promoter and subsequently transfected into amphotropic packaging cells (PA317)^22^,^23^. Infection of HOK cells by HSV-1 can be found *in vivo*, while HeLa cells represent a commonly used cell model for HSV-1 lytic infection^1^. Subsequently, HeLa and HOK cells were infected with these vectors and cell lines expressing these EGSs were screened out. No cytotoxicity was observed in the constructed cell lines for up to one month in MTT assays. Northern blot was used to detect EGS expression in these cells and only cell lines expressing similar EGS RNA levels were chosen for this study (Fig. 3, data not shown).

### EGS-mediated inhibition of HSV-1 ICP4 gene expression

**Figure 4:**
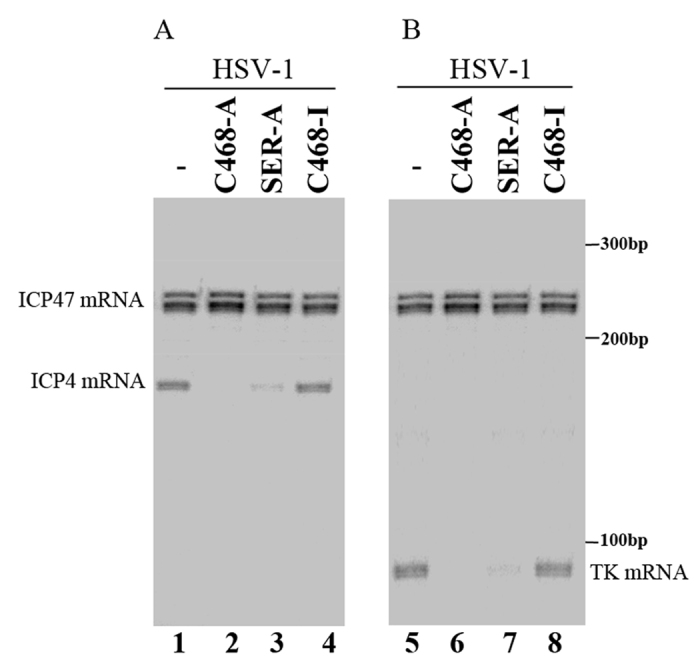
Expression levels of HSV-1 mRNAs with RNase protection assay. Parental HeLa cells (lanes 1 and 5), cell lines which expressed C468-A (lanes 2 and 6), SER-A (lanes 3 and 7), and C468-I (lanes 4 and 8) were infected with HSV-1(MOI = 0.9). Total RNAs were isolated from those cells 4 hours (**A**) or 10 hours (**B**) post infection. RNA samples (30 μg) were hybridized to the RNA probes containing the sequences of ICP4 and ICP47 (lanes 1–4) and to those containing ICP47 and TK mRNAs (lanes 5–8). The ICP47 mRNA was used as the internal loading control.

**Figure 5:**
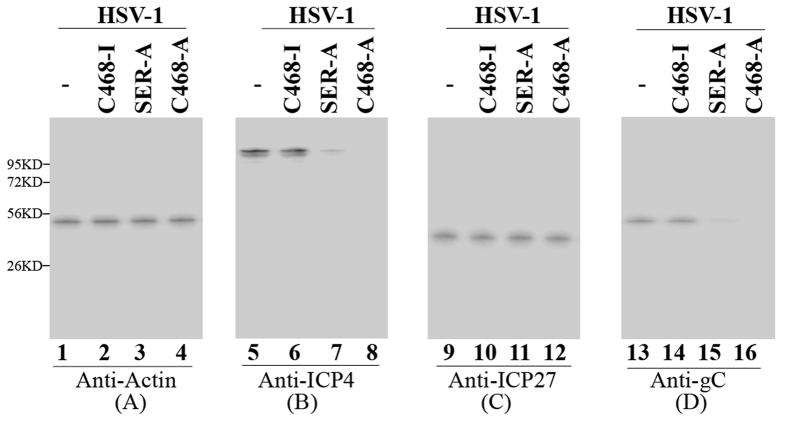
Expression levels of HSV-1 proteins with western blot analysis. Parental HeLa cells (lanes 1, 5, 9, 13), cell lines which expressed C468-I (lanes 2, 6, 10, 14), SER-A (lanes 3, 7, 11, 15), and C468-A (lanes 4, 8, 12, 16) were infected with HSV-1(MOI = 0.8). Protein samples were collected from those cells 12 hours post infection and detected with western blot analysis. Human actin (**A**), HSV ICP4 (**B**), ICP27 (**C**) and gC protein (**D**) were detected with specific antibodies.

To determine the EGS-mediated inhibition of ICP4 gene expression, cells were infected with HSV-1 (MOI = 0.05–1). Furthermore, some of cells were treated with 100 μg/ml cycloheximide which can inhibit protein synthesis and only allows the expression of viral IE transcripts but not early or late transcripts^1^. Total RNAs were then isolated. The expression levels of ICP4 mRNA were measured with an RNase protection assay and viral immediate early gene ICP47 mRNA was used as the internal loading control (Fig. 4). A decrease of ~96–97%, 75–77%, 8–9%, and 5–6% (results of experiments in triplicate) in the expression levels of ICP4 mRNA appeared in cells that expressed C468-A, SER-A, C-468-I, and SER-I, respectively (Tables 2 and 3). Meanwhile, the expression levels of ICP4 protein were assayed with western analysis using human actin as the internal loading control (Fig. 5). A reduction of 97%, 75–76%, 8%, and 5–7% (results of experiments in triplicate) in the expression levels of ICP4 protein appeared in cells expressing C468-A, SER-A, C468-I, and SER-I, respectively (Tables 2 and 3). Results of these two experiments implied that the specific cleavage of ICP4 mRNA induced by the EGSs might be the main cause contributing to the significant decrease of expression level of ICP4 in cells expressing C468-A or SER-A. The far less decrease in the expression level of ICP4 shown in cells expressing C468-I or SER-I was possibly because of the antisense effect as C468-I and SER-I shared the same antisense sequences of icp38 with C468-A or SER-A, but were not able to guide RNase P as the three nucleotides at the T-loop region were mutated (Fig. 1, Table 1). We detected no intracellular cleavage products of ICP4 mRNA, possibly due to the rapid degradation of these RNAs, which lacked either a poly A sequence or a 5′ cap structure.

### EGS-mediated inhibition of HSV-1 gene expression and growth

The ICP4 protein is one of the viral α or immediate early (IE) genes which is necessary for the activation of the expression of HSV early (β) and late (γ) genes^1^. The expressions of early and late genes of HSV are expected to be inhibited due to the repression of ICP4 expression. RNase protection and western blot analyses were applied to detect the expression of the TK mRNA (a β gene), ICP27 protein (an α protein), and glycoprotein gC (a γ protein), respectively (Figs 4 and 5). Significant decreases (75–97%) of the expression of TK and gC were detected in cells expressing C468-A or SER-A. However, no significant decrease was shown in cells expressing C468-I or SER-I, respectively (Figs 4 and 5). Similar results were also obtained when we assayed the protein expression level of HSV-1 capsid protein ICP35 and glycoprotein gB, two other γ gene products (Tables 2 and 3). Consistent with previous observations that ICP4 does not significantly affect viral IE expression^1^, no decrease in the level of ICP27 protein expression was found in cells expressing these EGSs (Fig. 5, lanes 9–12; Tables 2, 3). These results show that EGSs C468-A and SER-A specifically inhibit the expression of viral early and late genes but not immediate early (IE) genes.

**Figure 6:**
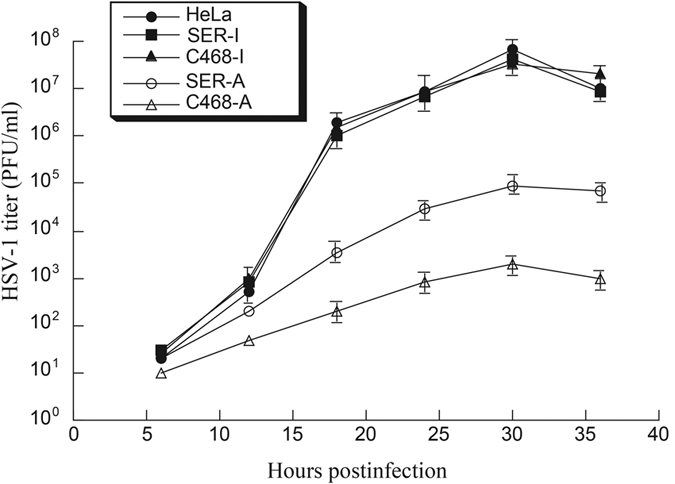
Growth analysis of HSV-1 in parental HeLa cells and cell lines expressing EGS RNAs. Equal number (5 × 10^5^) of parental HeLa cells and cell lines which expressed SER-I, C468-I, SER-A, and C468-A were infected with HSV-1 (MOI = 1). Virus stocks were prepared from the cells and culture media harvested at every 6 hours during 36 hours post infection. The virus titer of each sample was measured by counting the plaque forming unit (PFU) in Vero cells. The values are the average of three experiments, and the standard deviations are shown by the error bars.

**Figure 7:**
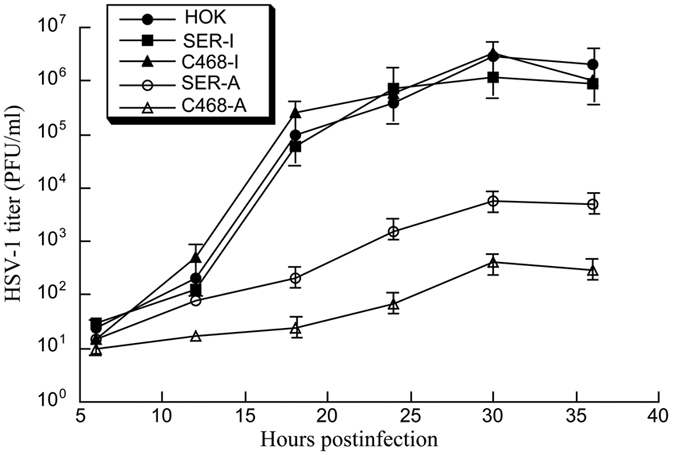
Growth analysis of HSV-1 in parental human oral keratinocytes (HOK) and HOK cell lines expressing EGS RNAs. Equal number (1 × 10^5^) of parental HOK cells and cell lines which expressed SER-I, C468-I, SER-A, and C468-A were infected with HSV-1 (MOI = 1.5). Virus stocks were prepared from the cells and culture media harvested at every 6 hours during 36 hours post infection. The virus titer of each sample was measured by counting the plaque forming unit (PFU) in Vero cells. The values are the average of three experiments, and the standard deviations are shown by the error bars.

The EGSs also seemed to inhibit viral growth in HSV-1 infected human cells. Equal number of parental HeLa and HOK cells, and cell lines that expressed SER-I, C468-I, SER-A, and C468-A were infected with HSV-1 (MOI = 1–1.5). Virus stocks were prepared from cells and culture media was harvested at every 6 hours during 36 hours post infection. The viral titer was measured by counting the number of plaque forming units (PFU) on Vero cells. After 18 hours post infection, a decrease of more than 7,000- and 500 folds in virus yield was observed in cells expressing C468-A and SER-A, respectively, compared to parental HeLa or HOK cells expressing no EGS (Figs 6 and 7). In contrast, no significant decrease was observed in those cells expressing C468-I and SER-I. These results support our observations that an inhibition of ICP4 expression in the presence of C468-A or SER-A leads to a significant decrease of expression of the β and γ genes of HSV.

## Discussion

EGS-mediated degradation of mRNA represents a new nucleic acid based approach for gene silencing applications. This strategy is a promising approach because intracellular RNase P can be guided with custom-designed EGSs to cleave any target mRNAs efficiently and specifically^3^,^24^. However, little is known about if EGS is effective in blocking HSV-1 growth by inhibiting the expression of viral essential genes. Our study presented here provides the direct evidence that engineered EGSs effectively block the replication and growth of HSV-1 in human cells.

It is still not clear how to improve the targeting activity of EGS and much work is needed to understand how to design EGSs exhibiting better efficacy in down-regulating gene expression in cultured cells. Here, making use of dimethyl sulphate (DMS), we determined an accessible region of HSV-1 ICP4 mRNA and constructed several EGSs that target the accessible region. Our results demonstrated that C468-A derived from ESG variant C468 was about 60 times more active in guiding RNase P to cleave target mRNA, more efficient (97% vs. 75%) in inhibiting ICP4 RNA and protein expression, and about 14 times more effective in reducing viral growth (~7000-fold vs. ~500 fold) than SER-A derived from natural pre-tRNA^SER^. In contrast, C468-I and SER-I, which share the similar structures with C468-A and SER-A respectively but with inactivated RNase P guiding activity because of mutations of the three conserved positions in the T loop, showed far less efficacy (less than 10%) in the inhibition of ICP4 mRNA and protein expression and exhibited no effect on reducing viral growth. The significant difference in the inhibition of ICP4 expression level and viral growth between C468-A and C468-I or between SER-A and SER-I indicates that the observed reduction is mainly caused by the specific EGS-mediated cleavage of ICP4 mRNA by RNase P rather than the antisense effect or other nonspecific effects of EGSs. The EGS (i.e. C468-A) showing higher activities in guiding RNase P to cleave the ICP4 mRNA sequence *in vitro* is also more effective in reducing HSV-1 gene expression and viral growth. These results are consistent with our observations that ESG-mediated inhibition of the expression level of ICP4, which is necessary for the expression of the early and late genes^1^, can result in inhibition of HSV-1 overall gene expression and viral growth. C468-A, which shows 60 folds higher activity to induce RNase P-mediated cleavage *in vitro* than SER-A, is also more effective in reducing ICP4 mRNA (97% vs. 75%) in cultured cells. These results suggest that the *in vitro* selection procedure may represent a good approach for the generation of highly effective EGSs for gene-targeting applications. The remaining 3% of the target mRNA in C468-A-expressing cells is possibly due to the expression of this little fraction of the mRNA in specific cellular compartments, which make it less accessible to be degraded by RNase P. Moreover, proper folding of the mRNA-EGS complexes in cells may also affect the efficacy of the EGS approach. Further studies will be needed to address these issues.

Enhanced stability of target mRNA-EGS complex seemed to be one of the main causes for the improved activity of EGS in guiding RNase P to degrade target mRNA. C468-A, which was 60 times more active in guiding RNase P to cleave icp38 sequence, showed 80 times greater affinity with icp38 sequence than SER-A (Table 1). It has been shown that the interaction between the variable and D-loops of pre-tRNA plays an important role in the constitution of the pre-tRNA tertiary structure and in the induction of RNase P-mediated cleavage^3^,^25^. In the icp38-C468-A complex, the 3′ region of icp38 is equivalent to the D-loop in a pre-tRNA (Fig. 1A,B,E). The difference between C468-A and SER-A is at their variable loop and T loop sequences. It is conceivable that the additional interactions between 3′ region of icp38 and the variable loop region of C468-A stabilize the mRNA-EGS complex and promote the targeting activity of the EGS. Thus, our study implies that *in vitro* selection is an effective approach for screening for highly active EGSs molecules.

In other studies, *in vitro* selection procedures have been widely used to generate functional RNA molecules such as ribozymes, aptamers, and EGSs which target mRNA encoding chloramphenicol acetyltransferase (CAT)^18^,^26^,^27^. In our studies, variant C468, which was selected to target a TK mRNA sequence^23^, was used to construct EGS C468-TK and C468-A that target two different mRNA sequences (i.e. TK and ICP4 mRNA). Our previous^23^ and current studies showed that C468-TK and C468-A are highly efficient in inducing RNase P-mediated cleavage of the TK and ICP4 mRNA sequences *in vitro*, respectively. It is conceivable that C468 could form unique tertiary interactions with both the targeted TK and ICP4 mRNA sequence in order to achieve efficient targeting activity. These results suggest that an EGS optimized for one target mRNA may exhibit efficient activity in targeting other mRNAs, possible by engineering EGS sequences to form novel and unique tertiary interactions with the targeted mRNA sequences. Further studies of EGS-based RNA interfering technology should improve the efficiency of EGS-mediated cleavage of target RNA by RNase P. These studies would facilitate the technology as a promising gene targeting approach for use in research and clinical therapeutic applications.

Induced cleavage of the ICP4 mRNA seems to be the main reason for the antiviral effect of EGSs. First, expression of the EGSs was not toxic to cells as cells expressing EGSs show no difference with parental cells expressing no EGSs in terms of cell growth and viability for up to one month (data not shown). Second, the antiviral effect (inhibition of viral growth and viral gene expression) associated with the expression of C468-A and SER-A appears to be caused by a reduction in the expression of ICP4. We observed overall reduction of viral early and late gene expression (e.g. TK, gC, ICP35, and gB) in cells which expressed C468-A and SER-A but not in those which expressed control C468-I or SER-I (Figs 4 and 5, Tables 2 and 3). The extent of inhibition of viral early and late gene expression and viral growth correlates with the extent of inhibition of the expression of ICP4 mRNA and protein. Third, designed ICP4-targeting EGSs specifically inhibit the expression level of ICP4 but not those of other immediate-early genes of HSV-1 like ICP27 and ICP47. No reduction was observed in the expression levels of ICP27 and ICP47 in the HSV infected cells expressing the EGSs (Figs 4 and 5, Tables 2 and 3). These results are consistent with the previous observations that ICP4 protein is required for overall expression of viral early and late genes and does not play a significant role in regulating the expression of viral IE genes^1^. Thus, induced cleavage of the ICP4 mRNA seems to be the main reason for the antiviral effect of EGSs.

In our previously studies, ribozymes derived from the catalytic RNA subunit of RNase P from *E.coli* were used to target TK and ICP4 mRNAs and were found to inhibit the expression of TK and ICP4 genes in cultured human cells^28^,^29^. Furthermore, the expression of anti-ICP4 mRNA ribozymes effectively blocked HSV-1 replication and infection in cultured cells^28^. In our current study, EGS RNAs, which are short RNA molecules, were constructed to bind to an ICP4 mRNA sequence and recruit endogenous RNase P to cleave the target mRNA. Thus, the EGS-based technology, which induces endogenous RNase P for cleavage, is unique and different from the ribozyme-based approach, which introduces an exogenous ribonuclease (i.e. RNase P-based ribozyme) for cleavage. Our results presented in this study provide direct evidence that EGSs are effective in blocking HSV-1 replication and infection in cultured human cells. The levels of EGS-mediated anti-HSV-1 efficacy are comparable to those observed using RNase P-based ribozymes^28^,^29^. These results further suggest that EGS may represent a novel class of antiviral gene-targeting agents.

Little is currently known about the stability and half-life of the EGSs in cells. It is conceivable that an EGS may be stable due to the possible association of the tRNA-like domains of the EGS with intracellular tRNA-binding proteins. Consistent with our previous studies detecting ICP47 mRNA^28^,^29^, two bands representing the RNase-protected products of the RNA probes hybridizing the ICP4 mRNA were detected in our RNase protection experiments (Fig. 4). It is possible that the RNase-protected products exhibited significant secondary and tertiary structures, which may lead to two different conformations that migrate differently in polyacrylamide gels even under denaturing conditions in the presence of urea. Further biochemical studies should be able to clarify this issue and reveal the nature of these two bands.

HSV-1 belongs to the human herpesvirus family that includes several other human viruses such as herpes simplex virus 2, varicella zoster virus, Epstein-Barr virus, cytomegalovirus, and Kaposi’s sarcoma-associated herpesvirus^1^. Like other herpesviruses, HSV-1 can engage in both lytic replication and latent infections. The ICP4 protein is one of the viral α or immediate early (IE) genes and a major transcription activator necessary for the expression of HSV early (β) and late (γ) genes^1^. Further study of EGS for anti-HSV application may focus on delivering EGSs into neuronal cells, where latent infection is established, and may determine if the delivered EGSs can prevent HSV-1 from reactivating from latent to lytic infection^1^. These studies will further promote the development of engineered EGSs for both basic research and clinical therapeutic applications.

## Methods

### Cells, viruses and antibodies

Human primary oral keratinocytes (HOK) were obtained from ScienCell Laboratories (Carlsbad, CA) and maintained according to the manufacture’s recommendation. Maintenance and culturing of Vero, HeLa, and PA317 cells (purchased from American Type Culture Collection, Manassas, VA) and infection and propagation of HSV-1 (strain F) in these cells were performed as described previously^20^,^30^. Monoclonal antibodies interacting with HSV-1 ICP4, ICP27, gB, and gC proteins were purchased from Virusyn (Taneytown, MD). The monoclonal antibody interacting with human actin was purchased from Sigma Inc (St Louis, MO). The monoclonal antibody MCA406, which interacts with HSV-1 ICP35 protein, was purchased from Bioproduct for Sciences Inc (Indianapolis, IN).

### Mapping of the accessible regions of ICP4 mRNA in HSV infected cells

HeLa and Vero cells infected with HSV-1 (MOI = 1) were incubated for 5–10 min with 5 mL of fresh culture media containing 1% DMS at 6 hours post infection^20^,^21^, then washed 3 times with 1 mM β –mercaptoethanol containing phosphate-buffered saline (PBS), and finally lysed by adding cell lysis buffer (10 mM Tris–HCl (pH 7.4), 150 mM NaCl, 1.5 mM MgCl_2_, 0.2% NP40)^20^,^21^. Total RNA was isolated from the lysates with phenol-chloroform extraction method. Primer extension assays to identify the DMS modification sites were then performed as described previously^20^,^21^.

### *In vitro* studies of EGSs and RNase P

SER-A sequence was amplified by PCR from plasmid pTK112^11^ (primers, 5′-GGAATTCTAATACGACTCACTATAGGTTAACgGTCGCGGGTGCGGTCTCCGCGC-3′ and 5′-AAGCTTTAAATGAGCCCAGCAGGATTTGAACCTGCGCGCGGAGACCGCAC-3′). C468-A sequence was amplified by PCR from plasmid pC468^19^ (primers, 5′-GGAATTCTAATACGACTCACTATAGGTTAACgGTCGCGGGCCACAGUUG–3′and 5′-AAGCTTTAAATGAGCCCAGTCGAACTCGAACGACCAACTGTGGC-3′). SER-I and C468-I sequences were derived from SER-A and C468-A sequence, respectively, and contained the three-nucleotide mutations (from 5′-TTC-3′ to AAG) in their T-loops (Fig. 1C–F). The 3′ primers for the construction of SER-I and C468-I were oligoSER-I3 (5′-AAGCTTTAAATGAGCCCAGCAGGATTTCTTCCTGCGCGCGGAGACCGCAC-3′) and oligoC468-I3 (5′-AAGCTTTAAATGAGCCCAGTCGAACTCCTTCGACCAACTGTGGC-3′), respectively. The substrate icp38 sequence was constructed by annealing oligonucleotide AF25 (5′-GGAATTCTAATACGACTCACTATAG-3′) and sICP (5′-CGGGATCCCTCGTCGCGGTCTGGGCTCGGGGTGGGCGCCTATAGTGAGTCGTATTA-3′).Constructed DNA sequences were used as templates to synthesize corresponding EGSs or substrate icp38 RNAs with T7 RNA polymerase. Human RNase P was extracted from HeLa cells as described previously^10^,^14^,^23^. Kinetic parameters were determined with previously described protocols^13^,^19^. Binding affinities were measured without the presence of RNase P^18^,^26^.

### Construction of EGS-expressing cells and detection of EGS expression

The procedures of constructing HeLa cells and human oral keratinocytes (HOK) which express different EGSs were modified from Miller and Rosman, as previously described^20^,^31^. Briefly, retroviral vectors containing different EGS sequences (LXSN-SER-A, LXSN-SER-I, LXSN-C468-A, and LXSN-C468-I) were transfected into amphotropic PA317 cells. Culture supernatants containing retroviral vectors were used to infect HeLa or HOK cells 48 hours post transfection. Cells were screened in media containing neomycin (600 μg/ml), and after two weeks the neomycin-resistant cells were isolated. Northern blot analysis was applied to determine the EGSs’ expression levels as described previously with an internal control of H1 RNA^20^,^31^.

### Infection of cells and detection of viral gene expression and growth

Equal number of Hela or HOK cells expressing different EGSs were either mock-infected or infected with HSV-1 with a MOI which is specified in the Results section. For detection of viral mRNA expression, total RNAs were extracted 4 or 10 hours post infection. For detection of viral protein expression, cell proteins were extracted 12 hours post infection. Some of the cells were treated with 100 μg/ml cycloheximide which can inhibit protein synthesis and then infected with HSV-1 to measure the levels of viral IE transcripts.

The RNA probes used to detect the ICP4 mRNA, ICP47 mRNA, and TK mRNA were synthesized from pICP4, pICP47 and pTK129, respectively. Antibodies used to detect human actin, ICP4, ICP27, ICP35, gB, gC were purchased from different companies, as described previously^28^,^32^. RNase protection assays and western blot assays were performed to detect the expression levels of viral mRNAs and proteins respectively, as described previously^28^,^32^.

To detect the level of the inhibition of viral growth, equal number of parental HeLa or HOK cells and cell lines which expressed SER I, C468-I, SER-A, and C468-A were infected with HSV-1 (MOI = 1–1.5). Virus stocks were prepared from the cells and culture media harvested at every 6 hours during 36 hours post infection. The virus titer of each sample was measured by counting the plaque forming unit (PFU) in Vero cells. The values presented are the average of three experiments.

## Additional Information

**How to cite this article**: Liu, J. *et al.* Inhibition of herpes simplex virus 1 gene expression and replication by RNase P-associated external guide sequences. *Sci. Rep.* **6**, 27068; doi: 10.1038/srep27068 (2016).

## Figures and Tables

**Table 1:** Overall cleavage rates (V_max(apparent)_/K_m(apparent)_)and binding affinities (K_d_) in EGS-mediated cleavage reactions of pre-tRNA^SER^ or ICP4 mRNA by RNase P.

**Substrate**	**K**_**m**_**(μM)**	**V**_**max (apparent)**_**(pmol·min**^**−1**^)	**V**_**max(apparent)**_**/K**_**m(apparent)**_**(pmol·μM**^**−1**^**·min**^**−1**^)	**K**_**d**_ **(μM)**
pre-tRNA^SER^	0.022 ± 0.071	0.069 ± 0.020	3.1 ± 0.5	
ICP4 RNA (icp38)				
+SER-A	0.60 ± 0.16	0.029 ± 0.010	0.048 ± 0.013	2.1 ± 0.5
+SER-I	ND	ND	<0.001	2.0 ± 0.5
+C468-A	0.29 ± 0.09	0.84 ± 0.36	2.9 ± 0.4	0.025 ± 0.005
+C468-I	ND	ND	<0.001	0.025 ± 0.005

Kinetic parameters were determined with previously described protocols^13^,^19^. Binding affinities were measured without the presence of RNase P. The values shown are the average derived from triplicate experiments. “ND”: not determined.

**Table 2:** Viral mRNA and protein expression in HeLa cells lines expressing EGS C468-A, C468-I, SER-A, and SER-I, respectively, presented as the percentages of inhibition compared to those in the parental HeLa cells.

**Viral gene**	**Gene class**	**HeLa**	**SER-I**	**C468-I**	**SER-A**	**C468-A**
ICP4 mRNA	IE	0%	6%	9%	77 ± 7%	97 ± 8%
ICP47 mRNA	IE	0%	0%	1%	1%	2%
TK mRNA	Early	0%	2%	2%	78 ± 6%	96 ± 9%
ICP4 protein	IE	0%	7%	8%	75 ± 7%	97 ± 8%
ICP27 protein	IE	0%	3%	3%	2%	1%
gC protein	Late	0%	1%	2%	77 ± 8%	95 ± 7%
ICP35 protein	Late	0%	0%	1%	77 ± 8%	95 ± 9%
gB protein	Late	0%	1%	2%	75 ± 7%	97 ± 8%

The values represent arithmetic means of triplicate experiments and the values of standard deviation which were less than 5% are not shown.

**Table 3:** Viral mRNA and protein expression in human oral keratinocyte (HOK) lines expressing EGS C468-A, C468-I, SER-A, and SER-I, respectively, presented as the percentages of inhibition compared to those in the parental human oral keratinocytes.

**Viral gene**	**Gene class**	**HOK**	**SER-I**	**C468-I**	**SER-A**	**C468-A**
ICP4 mRNA	IE	0%	5%	8%	75 ± 8%	96 ± 9%
ICP47 mRNA	IE	0%	1%	2%	1%	1%
TK mRNA	Early	0%	1%	1%	76 ± 8%	96 ± 9%
ICP4 protein	IE	0%	5%	8%	76 ± 8%	97 ± 9%
ICP27 protein	IE	0%	2%	2%	3%	2%
gC protein	Late	0%	2%	2%	76 ± 7%	97 ± 8%
ICP35 protein	Late	0%	1%	1%	76 ± 8%	95 ± 8%
gB protein	Late	0%	1%	1%	76 ± 8%	97 ± 9%

The values represent arithmetic means of triplicate experiments and the values of standard deviation which were less than 5% are not shown.
